# Relationship between caregiver characteristics and the reported quality of life of people with mild and moderate dementia

**DOI:** 10.15649/cuidarte.4646

**Published:** 2025-08-27

**Authors:** Mabel Tatiana Alvarez Polo, Rudy Marcela Sanchez Guarnizo, Duván Fernando Gómez Morales, Jasmin Bonilla-Santos

**Affiliations:** 1 Psychologist, Young Researcher, Master’s in Clinical Neuropsychology, Universidad Surcolombiana, Neiva, Colombia. E-mail: alvarezpolo2021@gmail.com Universidad Surcolombiana Neiva Colombia alvarezpolo2021@gmail.com; 2 Psychologist, Specialist in Clinical and Child Development, Master’s in Psychology, Master’s in Clinical Neuropsychology, Universidad Surcolombiana, Neiva, Colombia. E-mail: ruddysg@gmail.com Universidad Surcolombiana Neiva Colombia ruddysg@gmail.com; 3 Psychologist, Specialist in Applied Statistics, Neurocognition and Psychophysiology Laboratory, Universidad Surcolombiana, Neiva, Colombia. E-mail: duvanfgomezm@gmail.com Universidad Surcolombiana Neiva Colombia duvanfgomezm@gmail.com; 4 Psychologist, PhD in Cognitive Neuroscience, Psicosaberes Research Group, Universidad Cooperativa de Colombia, Neiva, Colombia. E-mal: jasminbonillasantos@hotmail.com Universidad Cooperativa de Colombia Neiva Colombia jasminbonillasantos@hotmail.com

**Keywords:** Informal Caregivers, Dementia, Caregiver Burden, Executive Function, Cuidadores Informales, Demencia, Carga del Cuidador, Función Ejecutiva, Cuidadores Informais, Demência, Carga do Cuidador, Função Executiva

## Abstract

**Introduction:**

Individuals with dementia are typically cared for at home by a family member who provides informal care, a role that can negatively affect their quality of life (QoL) and caregiving performance, thereby impacting the QoL of the person with dementia.

**Objective:**

To examine the relationship between caregiver characteristics and the QoL reported by individuals with mild or moderate dementia.

**Materials and Methods:**

This quantitative, cross-sectional, correlational study involved 50 dyads comprising individuals with mild to moderate dementia and their caregivers. The variables assessed were the QoL of the person with dementia, caregiver competence, caregiver burden, caregiver QoL, attention, cognitive flexibility, inhibitory control, decision-making, and working memory.

**Results:**

The study found significant correlations between caregiver competence, QoL, and burden/stress with the QoL of individuals with dementia. Executive function (inhibitory control and cognitive flexibility) was significantly associated with caregiver competence.

**Discussion:**

Caregiver QoL is positively associated with the QoL of individuals with dementia; therefore, enhancing caregiver-related characteristics contributes to effective disease management and the well-being of the person with dementia.

**Conclusion:**

The study demonstrates the impact of informal caregivers’ competence, burden, and executive function on the QoL of individuals with dementia.

## Resumen

**Introducción:**

Las personas que padecen demencia habitualmente son atendidas en casa por un familiar, quien realiza la labor de manera informal y que pueden verse perjudicados en su calidad de vida y en el desempeño de la actividad de cuidar e impactar en la calidad de vida de las personas con demencia.

**Objetivo:**

Examinar la relación de las características de los cuidadores sobre la calidad de vida informada por las personas con demencia en etapa leve o moderada.

**Materiales y métodos:**

Estudio de enfoque cuantitativo de diseño transversal de alcance correlacional. Los participantes fueron 50 díadas conformadas por personas con demencia leve y moderada y sus cuidadores. Las variables evaluadas fueron calidad de vida de la persona con demencia, competencia, sobrecarga, calidad de vida del cuidador, atención, flexibilidad cognitiva, control inhibitorio, toma de decisiones y memoria de trabajo.

**Resultados:**

El estudio evidenció correlaciones significativas entre la competencia, la calidad de vida y la sobrecarga/estrés del cuidador con la calidad de vida de las personas con demencia. La función ejecutiva (control inhibitorio y flexibilidad cognitiva) se asoció significativamente con la competencia de los cuidadores.

**Discusión:**

La calidad de vida del cuidador está relacionada positivamente con la calidad de vida de la persona con demencia, por lo que fortalecer las características del cuidador contribuye al adecuado manejo de la enfermedad y bienestar de la persona con demencia.

**Conclusiones:**

Se evidenció el impacto de la competencia, sobrecarga y función ejecutiva de los cuidadores informales sobre la calidad de vida de las personas con demencia.

## Resumo

**Introdução:**

Pessoas com demência geralmente são cuidadas em casa por um membro da família, que realiza esse trabalho informalmente. Isso pode afetar sua qualidade de vida e o desempenho das atividades de cuidado, impactando a qualidade de vida das pessoas com demência.

**Objetivo:**

Examinar a relação das características do cuidador com a qualidade de vida relatada por pessoas com demência leve e moderada.

**Materiais e métodos:**

Trata-se de um estudo quantitativo, transversal e correlacional. Os participantes foram 50 díades compostas por pessoas com demência leve e moderada e seus cuidadores. As variáveis avaliadas foram qualidade de vida da pessoa com demência, competência, sobrecarga, qualidade de vida do cuidador, atenção, flexibilidade mental, controle inibitório, tomada de decisão e memória de trabalho.

**Resultados:**

O estudo mostrou correlações significativas entre competência do cuidador, qualidade de vida e sobrecarga/estresse com a qualidade de vida de pessoas com demência. O funcionamento executivo (controle inibitório e flexibilidade cognitiva) foi significativamente associado à competência do cuidador.

**Discussão:**

A qualidade de vida do cuidador está positivamente relacionada à qualidade de vida de pessoas com demência; portanto, o fortalecimento das características do cuidador contribui para o manejo adequado da doença e o bem-estar das pessoas com demência.

**Conclusões:**

O impacto da competência, da sobrecarga e das habilidades executivas dos cuidadores informais na qualidade de vida de pessoas com demência foi evidente.

## Introduction

Globally, approximately 55 million individuals are living with dementia^1^. In Colombia, 108,259 individuals were reported to have been diagnosed with dementia in 2022^2^. Furthermore, the study titled *Prevalencia de deterioro cognitivo leve en regiones del sur de Colombia* (“Prevalence of Mild Cognitive Impairment in Regions of Southern Colombia”) reported high rates of mild cognitive impairment (MCI), with a prevalence of 51.9% in the province of Huila and 56.6% in Caquetá. At the regional level, the prevalence of MCI is as high as 53.6%. Moreover, the study estimated the conversion rate from MCI to dementia, projecting an annual probability between 10% and 15%, indicating that approximately one in four individuals with MCI may be at risk of developing dementia^3^. 

According to the World Health Organization, this constitutes a public health problem, as the demand for care increases, given that this disease follows a progressive and irreversible course, leading to dependence on a caregiver^1^. It is estimated that at least 80% of individuals diagnosed with dementia are cared for at home by a family member or friend who assumes the role of an informal caregiver^4^,^5^. While caregiving can be rewarding, it can also have detrimental effects on caregivers’ health, which is why they are often referred to as the “invisible second patients.”^6^,^7^ Caregivers experience substantial burden from the care they provide and may suffer from physical and psychological problems^8^. These challenges are negatively associated with their own quality of life (QoL) and the care provided to the person with dementia^9^. 

Caregivers possess varying levels of motivation, skills, and characteristics, all of which can influence their competence in performing caregiving activities. Managing the cognitive, behavioral, and psychological symptoms of dementia requires complex coping responses and should be distinguished from routine daily care^10^. Some caregiver characteristics that have been studied and documented include caregiver burden^11^,^12^, cognitive function, and QoL^13^,^14^. 

Caregiver burden refers to the emotional responses experienced by individuals who care for individuals with physical or mental disabilities.^15^ Caregiver burden may be perceived in situations where cognitive impairment is apparent^16^ or assessed objectively using standardized instruments^17^. Cognitive alterations reported among caregivers include impairments in working and declarative memory, attention, processing speed, inhibitory control, visuospatial skills, and executive function^13^,^14^,^18^. These deficits can significantly affect the well-being of people with dementia^19^. 

The influence of caregiver characteristics on the QoL of patients with dementia has been little studied. International studies have indicated that caregiver-related stress, social constraints, and perceived caregiving competence can contribute to reduced QoL, life satisfaction, and well-being in individuals with dementia^11^,^20^. Additionally, depression, caregiver burden, and poor overall health status among caregivers have been linked to higher levels of patient distress and inappropriate care interventions^21^. In Colombia, a study conducted in Bucaramanga, Santander, identified factors contributing to caregiver burden among informal caregivers of patients with Alzheimer’s disease. Increased burden was observed when patients exhibited more severe behavioral impairments, when caregivers held another job, and when they received little support from other family members^22^. Other studies have examined caregiver burden in the context of chronic diseases such as schizophrenia, cancer, diabetes, and hypertension, overlooking the impact on caregivers of individuals with dementia^23^. At the regional level, only one relevant study was identified. This study, conducted in Neiva, Huila, explored the relationship between caregiving competence and QoL among family caregivers of individuals with chronic noncommunicable diseases. Findings suggested that higher caregiving competence is associated with better QoL and reduced caregiver burden^24^. Despite growing interest in caregiver well-being, most studies have primarily focused on the impact of caregiving on the caregiver, with limited attention given to how caregiver competence and individual characteristics affect the QoL of individuals with dementia. 

The results of this study underscore the importance of caregivers’ cognitive and mental health, thereby fostering an understanding of how caregiving influences their lives^25^ and the QoL of individuals with dementia. The findings provide valuable insights for developing public policies, programs, and interventions aimed at safeguarding the mental health of informal caregivers. Accordingly, the objective of this study was to examine the relationship between caregiver characteristics and QoL reported by individuals with mild to moderate dementia. 

## Materials and Methods

This study employed a quantitative, cross-sectional, correlational design to assess the degree of association among multiple variables using statistical correlation techniques^26^. 

**Participants**

The study included dyads composed of individuals diagnosed with dementia and their informal caregivers residing in the municipality of Neiva, Huila. A non-probability convenience sampling method was employed to recruit participants^27^. A Structured Query Language query was conducted on the UROS Clinic database (UROSOFT V 2.0 system) to identify patients diagnosed with dementia and treated within the past 5 years. The retrieved data included basic demographic information, municipality of origin, type of care received, and primary admission diagnosis, categorized according to the International Statistical Classification of Diseases and Related Health Problems, 10th Revision (ICD-10). A total of 234 eligible cases were identified. Telephone outreach was conducted to contact potential participants, and inclusion and exclusion criteria were applied to both individuals with dementia and their respective informal caregivers. The inclusion criteria for individuals with dementia were as follows: community-dwelling status, clinical diagnosis of dementia (any subtype), and a Global Deterioration Scale (GDS) score between 4 and 5, indicating mild to moderate dementia^28^. After applying these criteria, a final sample of 50 caregiver–patient dyads (N = 50) was obtained. 

**Inclusion criteria for individuals with dementia**

- Prior clinical diagnosis of mild or moderate dementia (ICD-10) 

- Scoring at stages 4 or 5 on the GDS^28^

**Exclusion criteria for individuals with dementia**

- Presence of sensory disorders, particularly hearing impairment 

- Scoring <4 or >6–7 on the GDS^28^

**Inclusion criteria for informal caregivers**

- Agreeing to participate in the study and signing the informed consent form 

- Being the primary caregiver of an individual diagnosed with mild or moderate dementia 

- Not receiving financial compensation for caregiving activities 

- Residing in the city of Neiva 

- Providing care for at least 8 hours per day^29^

**Exclusion criteria for informal caregivers**

- Age below 18 years 

- Caregiving duration of less than 6 months^30^

- Having characteristics that prevent evaluation:

- Psychoactive substance use disorder

- Presence of any sensory, intellectual, neurological, or psychiatric disorder

**Operationalization of variables and instruments**

**Sociodemographic data:** Sociodemographic information was collected from individuals with dementia and their informal caregivers. The variables included age, gender, level of education, socioeconomic status, duration (in years), and intensity (average daily hours) of caregiving, among other relevant demographic indicators. 

***Variables and instruments for persons with dementia***

**Disease stage:** To classify the stage of dementia, the GDS was applied^28^. This scale delineates seven progressive stages of degenerative dementia: stage 1 (normal), stage 2 (age-associated memory impairment ), stage 3 (MCI), stage 4 (mild dementia), stage 5 (moderate dementia), stage 6 (moderately severe dementia), and stage 7 (severe dementia). The GDS demonstrated good internal consistency, with a reported Cronbach’s α of 0.82. 

**QoL of individuals with dementia:** Quality of life was treated as a nominal, qualitative, dependent variable. It was operationally defined as a multidimensional construct encompassing social, environmental, health-related, emotional, and spiritual domains. These include diverse elements such as occupational and leisure activities, hobbies, cognitive function, economic success, and psychological well-being. The instrument used to measure this variable was the QoL in Alzheimer’s Disease (QoL-AD). This instrument has two validated versions: one for the patient (QOL-ADp) and one for the caregiver (QOL-ADc) to assess the QoL of the person with dementia. Each version includes 13 items. The QOL-ADp scale had a Cronbach’s alpha of 0.88, and the QOL-ADc version had a Cronbach’s alpha of 0.82^31^. 

**Variables and instruments for assessing informal caregivers’ characteristics**

**Competence: **Caregiving competence was treated as an ordinal, qualitative, and independent variable. It was theoretically defined as “the capacity, ability, and preparedness of the person with a chronic illness and/or the family caregiver to conduct home-based caregiving activities.” This variable was assessed using the Home Care Competence Scale (GCPC-UN-CPC), family caregiver version. The instrument comprises 60 items distributed across six dimensions: knowledge, uniqueness or specific personal traits, procedural/instrumental skills, minimum conditions for caregiving or caregiver well-being, anticipation, and social relationships and interactions. The scale has a Cronbach’s α of 0.96^32^. 

**Caregiver QoL:** Quality of life was categorized as an ordinal, qualitative, and independent variable and defined as “a person’s perception of their position in life in the context of the culture and value systems in which they live and in relation to their goals, expectations, standards, and concerns.”^33^ The variable was measured using Item 1 of the WHOQOL-BREF questionnaire, which assesses general QoL. Responses were scored on a 5-point Likert scale ranging from 1 (very poor) to 5 (very good). The instrument has demonstrated satisfactory reliability, with a Cronbach’s α exceeding 0.80^34^. 

**Caregiver burden:** was considered an ordinal, qualitative, and independent variable, defined as the caregiver’s subjective experience of the demands associated with caregiving. The burden can be established objectively through the changes the caregiver makes in their life and the emotional impact caused by the demands of the caregiving role^35^. The Zarit Burden Interview, comprising 22 items, was used to assess this variable. The total scores were categorized as follows: no caregiver burden (≤46), mild caregiver burden (47–55), and severe caregiver burden (≥56). This scale has a Cronbach’s α of 0.92^36^. 

Attention, cognitive flexibility, decision-making, inhibitory control, and working memory were treated as ordinal, qualitative, and independent variables, with a mean of 50 and a standard deviation of 10. Scores were classified into the following performance levels: very low (3–10), low (11–25), average (26–75), and high (>75)^37^,^38^. Participants with the poorest performance were categorized as “very low,” while those with the strongest performance were categorized as “high.” 

**Attention** is both a mental process and a behavior, essential for discriminating, focusing on, processing, and monitoring relevant information^37^. Cognitive flexibility refers to the ability to shift thought or behavior in response to changing situational demands^39^. These two variables were assessed using the Trail Making Test (TMT-A and B), adapted for the Colombian population. TMT-A assesses attentional processes (R² = 0.426; p < 0.001). TMT-B evaluates executive functions, particularly cognitive flexibility (R² = 0.475; p < 0.001)^37^. 

**Decision-making** is defined as the ability to choose the most adaptive course of action by evaluating various behavioral alternatives. In this mental process, the cognitive aspects of the situation, the respective contingencies, and the emotional cues associated with the possible options are taken into account^40^. This variable was evaluated using the Modified Wisconsin Card Sorting Test (M-WCST) for the Colombian population. The version used comprised four stimulus cards and 48 response cards. The test showed statistically significant normative data (R² = 0.243; p < 0.001)^37^. 

**Inhibitory control** refers to the capacity to deliberately suppress dominant or automatic responses when the situation requires it^41^. This ability was assessed using the Stroop Color and Word Test, adapted for the Colombian population. The test includes 3 pages, each with 100 items arranged in 5 columns, to be read aloud from left to right within 45 seconds. Significant results were observed for Stroop Total Words (R2 = 0.381; p < 0.001), Stroop Total Colors (R2 = 0.397; p < 0.001), Stroop Words-Colors (R2 = 0.357; p < 0.001), and Stroop Interference (R2 = 0.58; p < 0.001)^37^. 

**Working memor**y is a temporary information storage and manipulation system^42^. This construct was measured using the Digit Span subtest of the Wechsler Adult Intelligence Scale-Fourth Edition (WAIS-IV). The subtest included three sections (Forward, Backward, and Sequencing), each with eight items and two trials (A and B). It has a Cronbach’s α = 0.93^38^. 

**Procedure**

The study was conducted over a period of 7 months and was structured into 3 distinct phases. In the first phase, individuals with a clinical diagnosis of mild or moderate dementia (within the past 5 years) and their informal caregivers were identified with the support of the UROS Clinic. Initial contact was established with potential participants, during which the project objectives were explained to them. Participants who met the established inclusion and exclusion criteria were invited to participate. GDS was administered to caregivers to confirm the dementia stage of the individual under their care. A contact database was subsequently compiled. In the second phase, an in-person appointment (lasting 45–60 minutes) was scheduled to obtain informed consent and administer scales and neuropsychological tests. In the third phase, the assessment instruments were scored, and individual evaluation reports were prepared and delivered to the participants. 

**Ethical considerations**

Ethical approval was granted by the Institutional Bioethics and Research Committee of the UROS SAS Clinic (Approval Minute No. 95). This study adhered to both national and international ethical guidelines for research involving human participants, in accordance with the Declaration of Helsinki. Participants’ rights and interests were prioritized throughout the study. All participants received comprehensive information regarding the study procedures, including their right to withdraw at any point. For individuals with dementia, their caregivers served as witnesses. 

**Data analysis**

Data were analyzed using the Statistical Package for the Social Sciences (SPSS), version 26.0. Descriptive statistics were applied to characterize the sample and summarize performance across all administered instruments. To assess the relationships between variables, bivariate correlations were computed using Spearman’s rank correlation coefficient (Spearman’s ρ)^43^. Correlation coefficients were interpreted based on both the direction and strength of the relationships. Positive correlations indicated that as one variable increased, so did the other, while negative correlations indicated that one variable increased as the other decreased. The strength of the correlations was interpreted according to the following ranges: -0.91 to -1.00 = perfect negative correlation; -0.76 to -0.90 = very strong negative correlation; -0.51 to -0.75 = strong negative correlation; -0.11 to 0.50 = moderate negative correlation; -0.01 to -0.10 weak negative correlation; 0.00 = no correlation; +0.01 to +0.10 = weak positive correlation; +0.11 to +0.50 = moderate positive correlation; +0.51 to +0.75 = strong positive correlation; +0.76 to +0.90 = very strong positive correlation; +0.91 to +1.00 = perfect positive correlation^43^. All data generated and analyzed in this study are publicly available in the Figshare repository^44^. 

## Results

The sample of individuals with dementia comprised 29 women (58.00%) and 21 men (42.00%). Among them, 7 participants (14.00%) were diagnosed with mild dementia, while 43 (86.00%) had moderate dementia. Participants’ ages ranged from 76 to 97 years, with 96.00% falling within this age range. Most had between 0 and 5 years of formal education (64.00%) and lived with a companion (98.00%) (Table 1). 

The caregiver group comprised 45 women (90.00%) and 5 men (10.00%). Half of the caregivers (50.00%) were between 28 and 59 years of age. Most belonged to a low socioeconomic stratum (64.00%) and had received 11 to 15 years of formal education (52.00%). In terms of caregiving experience, 68.00% reported providing care for 1 to 5 years. Additionally, 64.00% indicated that they provided care for 16 to 24 h per day (Table 2). 

**Table 1:** Sociodemographic data of individuals with dementia (n=50)

Sociodemographic Data	Frequency % (n)
Sex	
Female	58.00 (29)
Male	42.00 (21)
Age (years)	
Adults (55 and 59)	4.00 (2)
Older adults (≥60)	96.00 (48)
Years of receiving education	
0–5	64.00 (32)
6–10	10.00 (5)
11–15	18.00 (9)
16–20	8.00 (4)
Lives with a companion	
None	2.00 (1)
Family	98.00 (49)
Sensory deficits	
None	34.00 (17)
Decreased visual acuity	36.00 (18)
Decreased hearing	20.00 (10)
Both	10.00 (5)
Global detereortion scale	
Mild dementia	14.00 (7)
Moderate dementia	86.00 (43)

**Table 2:** Sociodemographic data of caregivers (n=50)

Domain	Frequency % (n)
Sex	
Female	90.00 (45)
Male	10.00 (5)
Age (years)	
Young (27)	2.00 (1)
Adult (28–59)	50.00 (25)
Older adults (≥60)	48.00 (24)
Years of receiving education	
1–5	6.00 (3)
6–10	14.00 (7)
11–15	52.00 (26)
16–20	26.00 (13)
≥21	2.00 (1)
Years dedicated to the care of the individual with dementia	
1–5	68.00 (34)
6–10	18.00 (9)
11–15	14.00 (7)
Hours of daily care	
6–10	16.00 (8)
11–15	20.00 (10)
≥15	64.00 (32)

Regarding the QoL of individuals with dementia, in the self-reported scale (QOL-ADp), 33 (66.00%) reported having QoL, whereas 17 (34.00%) reported not having QoL. In the caregiver-reported measure (QOL-ADc), 45 caregivers (90.00%) perceived that the person with dementia had QoL, and only 5 (10.00%) reported otherwise. 

Table 3 presents the distribution of caregiver competence levels across various domains. High levels of competence were observed in the domains of knowledge (n = 48; 96.00%), anticipation (n = 42; 84.00%), and procedural/instrumental skills (n = 47; 94.00%). Regarding overall caregiver QoL, 28 (56.00%) rated it as good to very good, while 10 (20.00%) reported poor to very poor QoL. 

In terms of caregiver burden, 28 (56.00%) experienced no burden, whereas 22 (44.00%) reported mild to severe burden. Evaluation of caregivers’ executive cognitive functions (attention, Cognitive flexibility, decision-making, working memory, and inhibitory control) revealed that most performed within the average range, except in decision-making, where lower performance was more prevalent: 20 caregivers (40.00%) scored low, and 18 (36.00%) scored very low. 

**Table 3:** Key Characteristic levels among informal caregivers of individuals with dementia (n=50)

Caregiver Characteristics	Subdomain -level	Frequency % (n)
Competence	Well-being	
	Medium	52.00 (26)
	High	48.00 (24)
	Uniqueness	
	Medium	52.00 (26)
	High	48.00 (24)
	Social interaction	
	Medium	66.00 (33)
	High	34.00 (17)
	Knowledge	
	Medium	4.00 (2)
	High	96.00 (48)
	Anticipation	
	Medium	16.00 (8)
	High	84.00 (42)
	Procedural/instrumental	
	Medium	6.00 (3)
	High	94.00 (47)
WHOQOL-BREF	Quality of life	
	Very poor	4.00 (2)
	Poor	16.00 (8)
	Normal	24.00 (12)
	Good	48.00 (24)
	Very good	8.00 (4)
ZARIT	Caregiver burden	
	No caregiver burden	56.00 (28)
	Mild caregiver burden	22.00 (11)
	Severe caregiver burden	22.00 (11)
Executive functioning		
Trail Making Test TMT-A	Attention	
	Low	40.00 (20)
	Average	58.00 (29)
	High	2.00 (1)
Trail Making Test TMT-B	Cognitive flexibility	
	Very low	2.00 (1)
	Low	16.00 (8)
	Average	76.00 (38)
	High	6.00 (3)
Stroop Interference	Inhibitory control	
	Very low	22.00 (11)
	Low	18.00 (9)
	Average	46.00 (23)
	High	14.00 (7)
Wisconsin	Decision-making	
	Very low	36.00 (18)
	Low	40.00 (20)
	High	24.00 (12)
Digit Span	Working memory	
	Very low	30.00 (15)
	Low	18.00 (9)
	Average	48.00 (24)
	High	4.00 (2)

Note: Home Care Competence Scale (GCPC-UN-CPC), World Health Organization Quality of Life (WHOQOL-BREF), ZARIT Burden Interview, Trail Making Test TMT-A, Trail Making Test TMT-B, Stroop Interference Test, Wisconsin, Card Sorting Test, Digit Span subtest.

To analyze the relationship between caregiver characteristics and the QoL of people with dementia, Spearman’s rank correlation coefficients (rs) were calculated: competence, caregiver burden, and QoL of individuals with dementia, both self-reported and caregiver-reported (Table 4). 

A strong positive correlation was found between caregivers’ well-being and their own QoL (rs = 0.544, p <0.01), caregiver-reported QoL of the individual with dementia (rs = 0.576, p <0.01), and self-reported QoL of the individual with dementia (rs = 0.609, p <0.01). In contrast, a strong negative correlation was observed between caregivers’ well-being and caregiver burden (rs = 0.626, p <0.01). 

In the uniqueness domain, a moderate positive correlation was found with caregivers’ QoL (rs = 0.418, p <0.01), caregiver-reported QoL of the individual with dementia (rs = 0.487, p <0.01), and self-reported QoL (rs = 0.351, p <0.05). A moderate negative correlation was also found between uniqueness and caregiver burden (rs = –0.435, p <0.01). The social interaction domain exhibited strong positive correlations with caregiver-reported QoL (rs = 0.606, p <0.01) and self-reported QoL (rs = 0.601, p <0.01), and a strong negative correlation with caregiver burden (rs = 0.588, p <0.01). 

The knowledge domain demonstrated moderate positive correlations with caregiver-reported QoL of the individual with dementia (rs = 0.483, p <0.01) and self-reported QoL of the individual with dementia (rs = 0.333, p <0.05), and a moderate negative correlation with caregiver burden (rs = –0.404, p <0.01). 

Anticipation showed a moderate positive correlation with caregiver-reported QoL of individuals with dementia (rs = 0.363, p <0.01) and a strong negative correlation with caregiver burden (rs = –0.523, p <0.01). In the procedural/instrumental domain, a strong positive correlation was found with caregiver-reported QoL (rs = 0.534, p <0.01). 

Caregivers’ overall QoL was negatively correlated with caregiver burden (rs = –0.649, p <0.01), while caregiver burden was also strongly negatively correlated with caregiver-reported QoL of individuals with dementia (rs = –0.641, p <0.01). 

 Finally, self-reported QoL of individuals with dementia showed moderate positive correlations with caregivers’ QoL (rs = 0.441, p = 0.001) and caregiver-reported QoL of the individual with dementia (rs = 0.424, p <0.01), as well as a moderate negative correlation with caregiver burden (Table 4).

**Table 4:** Correlations between caregivers’ characteristics and the quality of life of individuals with dementia

Variables	1	2	3	4	5	6	7	8	9	10
1. Well-being	—	—	—	—		—	—	—	—	—
2. Uniqueness	0.504**	—	—	—	—	—	—	—	—	—
3. Social interaction	0.567**	0.547**	—	—	—	—	—	—	—	—
4. Knowledge	0.360*	0.560**	0.475**	—	—	—	—	—	—	—
5. Anticipation	0.284*	0.569**	0.388**	0.672**	—	—	—	—	—	—
6. Procedural/instrumental	0.380**	0.558**	0.492**	0.716**	0.577**	—	—	—	—	—
7. Caregivers’ QoL	0.544**	0.418**	0.488**	0.265	0.240	0.180	—	—	—	—
8. Caregiver burden	−0.626**	−0.435**	−0.588**	−0.404**	−0.523**	−0.369**	−0.649**	—	—	—
9. Caregiver-reported QoL of the individual with dementia	0.576**	0.487**	0.606**	0.483**	0.363**	0.534**	0.474**	−0.641**	—	—
10. Self-reported QoL of the individual with dementia	0.609**	0.351*	0.601**	0.333*	0.265	0.373**	0.441**	−0.395**	0.424**	—

Note: **p=<0,01; *p=<0.05.

Table 5 presents the correlations between caregivers’ executive functions, their caregiving competence, and the QoL reported by individuals with dementia. Cognitive flexibility showed a moderate positive correlation with the uniqueness domain of caregiver competence (rs = 0.315, p <0.01). Similarly, inhibitory control demonstrated moderate positive correlations with the uniqueness domain (rs = 0.384, p <0.01), knowledge (rs = 0.316, p <0.05), anticipation (rs = 0.293, p <0.05), and procedural/instrumental (rs = 0.493, p <0.01) domains. However, no statistically significant correlations were observed between caregivers’ executive functions and the QoL reported by individuals with dementia. 

**Table 5:** Correlation between caregivers’ executive functions and competence and the QoL of individuals with dementia

Variables	Well-being	Uniqueness	Social Interaction	Knowledge	Anticipation	Procedural	QP
Attention	−0.014	0.261	0.040	0.271	0.257	0.210	0.080
Cognitive flexibility	0.155	0.315*	0.107	0.156	0.134	0.169	0.033
Inhibitory control	0.152	0.384**	0.201	0.316*	0.293*	0.493**	0.147
Decision-making	0.091	0.063	0.152	−0.081	−0.260	−0.096	0.017
Working memory	0.082	0.081	0.189	−0.058	0.025	0.148	−0.003

Note: Home Care Competence Scale (GCPC-UN-CPC), Trail Making Test TMT-A, Trail Making Test TMT-B, Stroop Color and Word Test (interference), Wisconsin Card Sorting Test, Digit Span subtest, and QoL of the person with dementia (QP). *p ≤ 0.05.**p ≤ 0.01.

## Discussion

This study explored the relationship between informal caregivers’ characteristics and the QoL reported by individuals with mild to moderate dementia. Significant positive correlations were identified between caregivers’ QoL and their competence in the domains of well-being, uniqueness, social interaction, knowledge, and procedural/instrumental skills. Conversely, a negative relationship was observed between caregivers’ burden levels and the QoL reported by individuals with dementia. Although no significant associations were found between caregivers’ executive functions and the QoL of individuals with dementia, positive correlations were observed between inhibitory control and competence (more specifically, the uniqueness, knowledge, anticipation, and procedural/instrumental domains). Additionally, cognitive flexibility was positively correlated with the uniqueness domain. 

Demographically, the majority of caregivers in this study were older adult women with a relatively high level of education who cohabited with the person with dementia. These findings align with previous literature reporting similar caregiver profiles^9^,^19^,^45^-^49^. Notably, the caregivers’ average educational attainment exceeded 8 years, which may have positively influenced their performance on cognitive tasks. This level of education is considered high in other studies^50^,^51^, and evidence suggests that the higher the caregiver’s educational attainment, the better their perception, coping skills, and adaptation to the demands of caregiving^52^,^53^. In contrast, other studies have found that caregivers with lower educational levels tend to acquire less knowledge about the disease and report higher levels of caregiver burden when providing care^53^-^54^. These observations highlight that both educational level and average performance on cognitive assessments may act as protective factors that enhance the quality of care provided to individuals with dementia. 

These findings underscore the importance of integrating the sociodemographic characteristics of caregivers into the design of supportive policies and intervention programs. Factors such as gender, age, relationship to the care recipient, and education level can influence the experience and needs of caregivers of individuals with dementia. Additionally, existing research has pointed out that caregivers, being family members, often view their role as a responsibility or an opportunity to strengthen their relationship with the individual with dementia, which in turn can reduce caregiver burden and alleviate behavioral symptoms of dementia^55^,^56^. 

In the present study, the QoL of individuals with dementia did not appear to be adversely affected. This may be attributed to the caregivers’ sociodemographic characteristics and adequate levels of competence they reported. The results of this study showed significant correlations between caregiver characteristics and the QoL of individuals with dementia. The main caregiver characteristics associated with the QoL of the person with dementia are discussed below. 

Caregivers’ well-being demonstrated a significant positive association with QoL of individuals with dementia, both caregiver-reported and self-reported. Other studies^19^,^57^ suggest that the emotional and physical well-being of caregivers has a direct impact on the perceived QoL of the person with dementia. Moreover, caregivers with higher well-being are better prepared to provide more effective care based on their knowledge of the disease and its progression, which in turn benefits the patient’s QoL^11^. Evidence shows that when caregivers can identify the positive aspects of caregiving, their mood and overall well-being improve,^11^ which in turn enhances their caregiving role and positively influences the QoL of individuals with dementia. As previously discussed, caregiving can be both a rewarding and a stressful experience, potentially compromising caregivers’ health and well-being^8^. However, it is also important to acknowledge that caregivers may experience positive relational aspects that favorably influence their well-being and, consequently, their caregiving role. Research has shown that perceived positive aspects of caregiving are associated with better QoL^58^ and well-being^19^,^56^. 

A positive relationship between the uniqueness or individuality domain and QoL of individuals with dementia, in both the caregiver-reported and self-reported measures, was observed. Recognizing and respecting the individuality of a person with dementia promotes personalized and respectful care, contributing to their QoL. Given the degenerative nature of dementia, the caregiver-patient relationship dynamics inevitably evolve, with caregivers taking on their role as the person with dementia becomes more dependent, requiring both to adapt to the diagnosis and the changes brought about by the disease^11^. Therefore, it is essential that caregivers receive accurate, tailored information, particularly in the early stages of the disease and throughout its progression^59^. 

Regarding social interaction, activities that foster socialization can significantly contribute to improving the caregiver’s QoL and, in turn, the QoL of the person with dementia. Research has shown that greater caregiver social participation is associated with increased involvement of individuals with dementia in activities such as visiting relatives, attending religious services, and volunteering^60^. Similarly, having fewer social restrictions, resulting from receiving more support in caregiving, can reduce caregivers’ stress levels and, consequently, positively impact their well-being and the level of care they provide. Conversely, when caregivers receive no assistance, the person with dementia may be affected, as the caregiver’s well-being is likely to be compromised^11^. 

Regarding the knowledge domain, greater knowledge of the disease and its management can translate into more effective care strategies, thereby benefiting the QoL of individuals with dementia^11^,^61^,^62^. In particular, being informed about available formal and informal support services, best caregiving practices, communication techniques, and ways of addressing the challenges that arise throughout the progression of the disease are aspects that can help family caregivers develop effective coping strategies^59^,^63^. 

In terms of anticipation, the caregiver’s ability to anticipate the patient’s needs can help improve daily caregiving experiences. By focusing on promoting relationships, encouraging social participation, and facilitating daily functioning, caregivers not only address physical and mental health issues but also ensure high-quality care, thereby contributing to improved QoL for people with dementia^64^. 

Similarly, adequate caregiver procedural and instrumental competence—such as managing daily routines and administering medication— is fundamental to maintaining and improving the patient’s QoL. Therefore, training caregivers about the disease and the importance of medication adherence can help ensure optimal treatment^61^,^65^. 

Finally, this study confirmed a significant negative correlation between caregiver burden/stress and the QoL of individuals with dementia. High levels of stress and burden among caregivers can lead to less optimal care, reduced quality of life, and poorer interactions between the caregiver and the person with dementia^11^,^56^. Research has shown that, particularly in challenging times, positive and negative psychological states often coexist. Positive emotions play a vital role in understanding how individuals respond and adapt to such demanding experiences. As such, strengthening caregivers’ sense of competence is essential for reducing stress levels^56^. This is particularly important given that higher stress levels have been associated with lower self-evaluations of QoL by individuals with dementia^11^,^66^. 

Evidence shows that the QoL of people with dementia influences the QoL of caregivers, and vice versa. Thus, one of the main objectives in dementia care should be the preservation of caregivers’ QoL across multiple dimensions, as this can directly improve disease management and ultimately enhance the QoL of the person with dementia^9^. In line with previous findings, this study also identified that lower caregiver burden is generally associated with better self-assessed QoL by the person with dementia, and vice versa^11^. Similar results were found in another study, where caregiver burnout and caregiver burden negatively correlate with caregivers’ QoL^67^. 

Another critical factor influencing caregivers’ QoL is their executive functioning. In this study, inhibitory control showed positive correlations with competence in the domains of uniqueness, knowledge, anticipation, and procedural/instrumental skills, while cognitive flexibility was positively associated with uniqueness. These findings contrast with previous research reporting impairments in inhibitory control, attention, working memory, declarative memory, episodic memory, cognitive flexibility, and processing speed among caregivers^13^,^18^,^68^-^70^, which were found to be negatively associated with the QoL of the person with dementia. 

Generally, caregivers who possess knowledge about dementia, its treatments, and other related aspects report less uncertainty, experience reduced caregiver burden, and demonstrate improved QoL. This knowledge enables them to anticipate the needs of the person with dementia, allowing caregivers to provide more efficient and detailed care^15^,^71^. Similarly, caregivers who report stronger skills and caregiving abilities also tend to report higher QoL^24^. In contrast, other studies have found that many caregivers report medium to low competence in various domains, highlighting the need to investigate caregiver training processes as a determining factor in the quality of care provided^32^,^72^. Importantly, this study found that the well-being domain was negatively correlated with caregiver burden, suggesting that caregivers who perceive themselves as having higher well-being also report lower levels of stress and burden. Supporting this, research indicates that caregivers’ stress perceptions can have a detrimental impact on their well-being^19^. These findings align with our data, where 56% of caregivers reported no caregiver burden and 22% reported only mild caregiver burden, suggesting that most caregivers in this sample experienced relatively low stress levels. This finding is consistent with prior studies which have shown low levels of caregiver burden, even among those providing more than 200 hours of care per month^48^. 

Taken together, these results highlight the importance of multidimensional support for caregivers, including reducing caregiver burden, promoting well-being, enhancing knowledge about the disease, and facilitating meaningful social engagement. Strengthening these areas not only improves the caregivers’ QoL but also improves te QoL of individuals with dementia. Finally, as dementia is a progressive neurodegenerative disorder, the role of caregiving evolves over time, often leading to fluctuations in caregivers’ QoL^19^. Therefore, continuous and adaptable support for caregivers is essential for mitigating stress and strengthening their feelings of competence, generating a positive impact for both the caregiver and care recipient^11^,^19^. 

**Study limitations and future perspectives**

This study has several limitations that should be acknowledged. First, the sample size was a limitation. A larger sample could have helped draw more definitive conclusions regarding the identified relationships. Additionally, the use of convenience sampling may have introduced selection bias. Future research should aim to re-examine this issue to obtain more information on the relationship between the QoL of people with dementia and the characteristics and role of the caregiver. Another limitation to consider is the study design, which was descriptive and cross-sectional and therefore did not allow for causal inferences. Furthermore, the use of a convenience sampling technique prevents generalizability of the results. Despite these limitations, the present study underscores the significant influence of caregivers’ QoL and competence on the QoL of individuals with dementia. Specifically, caregiver competence appears to be a key factor in preventing caregiver burden. In turn, caregiver burden is associated with cognitive decline in caregivers and impacts the care they provide. 

## Conclusions

This study contributes to a deeper understanding of how caregivers’ characteristics and competence influence the QoL of individuals with dementia. The findings highlight that caregivers’ competence levels were generally moderate to high. The dimensions of social interaction, uniqueness, and well-being were rated at a medium level, reflecting the essential skills needed to address the daily challenges of caregiving. 

In contrast, knowledge, anticipation, and procedural/instrumental dimensions were rated highly, suggesting that many caregivers may have received psychological education or training on managing this neurodegenerative disease. Regarding the QoL of individuals with dementia, both self-reported and caregiver-reported measures yielded positive outcomes. Notably, few caregivers reported experiencing severe caregiver burden, which is known to be negatively associated with caregivers’ QoL. 

Significant relationships were observed between the QoL reported by individuals with dementia and caregiver-related variables, including caregiver burden, QoL, and competence in the domains of well-being, uniqueness, social interaction, knowledge, and procedural/instrumental skills. In terms of executive functioning, inhibitory control was positively correlated with caregiver competence across uniqueness, knowledge, anticipation, and procedural/instrumental dimensions. Cognitive flexibility was also positively associated with the uniqueness domain. However, no direct association was identified between caregivers’ executive functioning and the QoL of individuals with dementia. 

These results underscore the impact of caregivers’ characteristics on the QoL of individuals with dementia. They offer valuable insights into the components that should be incorporated into interventions to achieve a greater impact on people with dementia. This study underscores the need for research and interventions that target both individuals with dementia and their informal caregivers. While many studies focus on only one group, our findings demonstrate that supporting both members of the care relationship can lead to more effective and efficient interventions. 
